# The Iowa Gambling Task in depression – what have we learned about sub-optimal decision-making strategies?

**DOI:** 10.3389/fpsyg.2013.00732

**Published:** 2013-10-10

**Authors:** Anita Must, Szatmar Horvath, Viola L. Nemeth, Zoltan Janka

**Affiliations:** ^1^Department of Psychiatry, University of SzegedSzeged, Hungary; ^2^Department of Psychiatry, Vanderbilt UniversityNashville, TN, USA

**Keywords:** decision-making, depression, anhedonia, habenula, vmPFC, outcome

## Abstract

Our earlier study found patients with depression to show a preference for larger reward as measured by the Iowa Gambling Task (IGT). In this IGT version, larger rewards were associated with even larger consequent losses. In the light of the clinical markers defining depressive disorder, this finding might appear controversial at first. Performance of depressed patients on various decision-making (DM) tasks is typically found to be impaired. Evidence points toward reduced reward learning, as well as the difficulty to shift strategy and integrate environmental changes into DM contingencies. This results in an impaired ability to modulate behavior as a function of reward, or punishment, respectively. Clinical symptoms of the disorder, the genetic profile, as well as personality traits might also influence DM strategies. More severe depression increased sensitivity to immediate large punishment, thus predicting future decisions, and was also associated with higher harm avoidance. Anhedonic features diminished reward learning abilities to a greater extent, even predicting clinical outcome. Several questions about how these aspects relate remain to be clarified. Is there a genetic predisposition for the DM impairment preceding mood symptoms? Is it the consequence of clinical signs or even learned behavior serving as a coping strategy? Are patients prone to develop an aversion of loss or are they unable to sense or deal with reward or the preference of reward? Does the DM deficit normalize or is a persisting impairment predictor for clinical outcome or relapse risk? To what extent is it influenced by medication effects? How does a long-lasting DM deficit affect daily life and social interactions? Strikingly, research evidence indicates that depressed patients tend to behave less deceptive and more self-focused, resulting in impaired social DM. The difficulty in daily interpersonal interactions might contribute to social isolation, further intensifying depressive symptoms.

## INTRODUCTION

Depression is traditionally considered an affective disorder. Yet, research in the past decades has drawn attention to the substantial impairment in cognitive function. Various aspects of cognitive disturbance have frequently been reported in the acute phase of the illness (Harvey et al., 2004; Rogers et al., 2004). These include domains of executive function, such as planning and problem solving (Naismith et al., 2003), inhibition and semantic fluency (Ravnkilde et al., 2002; Gohier et al., 2009) – present even in first episode major depressive disorder (Schmid and Hammar, 2013) – decision-making (DM; Chamberlain and Sahakian, 2006) and various aspects of memory processes (Rose and Ebmeier, 2006; Taylor Tavares et al., 2007). Convincing research evidence has accumulated about the key cognitive deficits characterizing a major depressive episode (for a review and meta-analysis see Castaneda et al., 2008; Lee et al., 2012). The cognitive alterations affect several aspects of daily life functioning including work performance, planning and DM and even social interactions. However, the cognitive profile defined during a depressive episode might not merely be the consequence of depressive symptoms (Hammar and Ardal, 2009). Findings indicate that improvement of the cognitive disturbance and aspects of daily life functioning are not always in accordance with the remission of a depressive episode (Kennedy et al., 2007). Nevertheless, the cognitive deficit plays a crucial role in functional recovery from depression (Jaeger et al., 2006) while a persistent cognitive impairment might be an important factor associated with long-lasting disability in everyday functioning. In his thought provoking review, Kendler offers the concept that our own decisions might well intervene in causal pathways from the genome to behavior and phenotype. Kendler argues, that human cognitive DM capacity may either suppress or augment the expression of risk genes and heritability of a trait (Kendler, 2013). Consequently, the DM capacity might have a fundamental effect on social skills and coping strategies, influencing vulnerability, preventing symptoms or even enhancing relapse risk.

The Iowa Gambling Task (IGT) has had a substantial impact on our understanding of the complex aspects of DM in the past two decades. Here we aim to provide a targeted review of the literature in the effort to shed some light on its revealing role on the DM deficit in depression. Special emphasis is directed to the influence of anhedonic symptoms, the role of habenula and the ventromedial prefrontal cortex (vmPFC) in dysfunctional DM and their combined effect on outcome prediction.

## THE IOWA GAMBLING TASK – A BENCHMARK OF REAL-LIFE DECISION-MAKING

Designed by Bechara et al. (1994) the IGT resembles the real-life DM process relying on contingencies of reward and penalty by taking the advantage of the uncertainty of outcomes. Development of the task was guided by the somatic marker hypothesis assuming that signals of the body given as a reaction to the experience of reward or punishment guide behavior toward long-term beneficial choices (Damasio, 1994). The IGT involves four decks of cards and participants are asked to freely choose one card at a time from one of the decks. In the original version (“ABCD”) selection from two decks (A and B) is followed by a high immediate reward in measures of play money on a computerized system, but at unpredictable points, an even higher penalty occurs. Picking from the other two decks (C and D) is associated by smaller gain but even smaller future loss, which proves to be more advantageous in long-term. Thus, participants start to show a preference for the more advantageous decks of cards and tend to avoid decks A and B, defined by disadvantageous future consequences. This DM tendency is also predicted by anticipatory skin conductance responses among healthy participants. Driven by the interest to understand the neuroanatomical background and the motivational aspects of the DM process, Bechara et al. (2000) designed variant (“EFGH”) of the original IGT version by reversing the order of reward and punishment. Here the advantageous decks (E and G) yielded immediate high loss but even higher consequent gain, while decks F and H contained the more disadvantageous cards on long-term with smaller penalties but even lower rewards at unforeseeable time points (Bechara et al., 2000). Patients suffering from major depressive disorder are typically found to show altered sensitivity to reward and punishment on both IGT variants. This involves fewer selections from the advantageous decks on the “ABCD” version (Han et al., 2012) and less shifting of DM strategies in the light of encountered experiences during both the standard and the contingency-shift phases of the IGT (Cella et al., 2010). Our earlier study detected a preference for larger reward as measured by IGT in a group of depressed patients. While performing the “ABCD” version, participants suffering from depressive disorder tended to choose from the disadvantageous decks offering high immediate reward. Despite the consequent increased punishment, patients failed to shift strategy and to develop a long-term beneficial DM tendency (Must et al., 2006). Increased reward preference in depression might appear controversial at first. However, the critical underlying factor might rather be the impaired ability in reinforcement processing. The reward-related processing deficit revealed in depression leads to a difficulty to integrate feedback information in guiding future behavior. Consequently, depressed patients focus on the immediate outcome thus preferring the decks with higher reward on the short-term, The decreased ability to integrate reward-related reinforcement history might thus be considered a manifestation of reduced reward responsiveness (Eshel and Roiser, 2010). Depressed patients appear to experience a more pronounced decisional conflict in DM situations explained by a dysfunctional processing of seemingly unpredictable or counterfactual outcomes (Chase et al., 2010). Moreover, depressed patients have been characterized by a prolonged attenuation of temporal discounting of rewards (Lempert and Pizzagalli, 2010) also suggesting the impairment of DM processing. Considering the difficulty to shift strategy even after encountering large subsequent penalties we might even speculate that depressed patients consider the loss to be inevitable, inherent to a rewarding stimuli. Findings suggest that depressed individuals presume punishing consequences to be more likely to occur than rewarding ones. This is supported by the notion that depressed patients did not change their behavior under conditions of absent versus negative feedback, seemingly expecting some penalty, as if predestinated (Elliott et al., 1998). If faced with immediate punishment, as in the “EFGH” version of the IGT, a potential large gain in the future might not outweigh a high loss in the present. In this case individuals with depression might prefer to make fewer selections of the risky decks, picking more cards defined by low magnitude punishment (Cella et al., 2010) though disadvantageous on the long-term. Strikingly, acutely depressed patients have also been shown to learn to avoid risky responses better than controls (von Helversen et al., 2011). This might be related to higher harm avoidance, enhanced sensitivity to aversive stimuli, a bias toward negative self-evaluation and is also consistent with clinical symptoms of depression (Paulus and Yu, 2012). The possibility might even be raised that certain subgroups of depressed patients with different leading clinical symptoms are characterized by distinct DM strategies. In the next section we discuss factors potentially influencing the DM process including clinical markers and neuroanatomical correlates of depression. A special emphasis is given to the role of DM strategies based on different aspects of reward contingencies in predicting social and functional outcome of the disorder.

## DECISION-MAKING IN DEPRESSION

### THE INFLUENCE OF CLINICAL SYMPTOMS: ANHEDONIC vs. NON-ANHEDONIC PATIENTS

The effect of depressive symptoms on the DM process constitutes an area of particular interest but not only in association with illness state, i.e., acute phase or remission. Converging evidence examining cognitive disturbances in the longitudinal course of depression suggests that certain neuropsychological domains are more related to the clinical state than others. Among latter, the deficit in executive function and attention might constitute the most trait-like impairment (Douglas and Porter, 2009). Neurocognitive alterations involving executive functions are present in groups of depressed adolescents (Maalouf et al., 2011) and can be detected in unmedicated patients with major depressive disorder (Porter et al., 2003). Disturbance of the complex construct of DM might also be present before the onset of depressive symptoms and contribute to their persistence. A mechanism of critical importance implied in this process is reduced reward learning. Depressed patients tend to show a difficulty in modulating behavior as a function of reward (Elliott et al., 1996). Evidence suggests, that this impairment is particularly associated with anhedonia. Anhedonia is defined as the inability to experience pleasure, to respond to positive reinforcers resulting in dysfunctional DM and consequently in an impairment in goal-directed behavior (Der-Avakian and Markou, 2012). Recent evidence indicates that depressed patients with anhedonic symptoms are characterized by a significant deficit in reward learning abilities. Moreover, anhedonic features not only influenced behavioral modulation in the light of reward contingencies, but were found to have a predictive role for the diagnosis of major depression to persist for at least 8 weeks besides antidepressive treatment (Vrieze et al., 2013). This raises the notion of an interaction between a persistent DM deficit and symptoms of anhedonia in depression serving as predictors of clinical outcome. In the past decades, the IGT has proven an effective method to address this trait-like DM disturbance.

### THE NEUROANATOMICAL BACKGROND: A FOCUS ON INTERACTIONS OF THE HABENULA AND THE vmPFC

**FIGURE 1:**
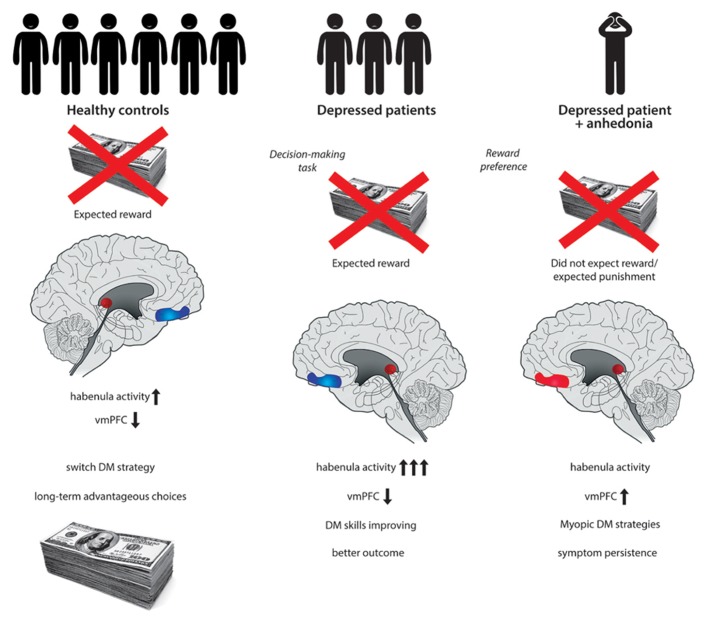
**After encountering a large penalty eventually exceeding an expected reward while playing the Iowa Gambling Task (IGT), healthy control participants tend to switch strategy.** The absence of an expected reward is associated with habenula activation and subsequent decrease in ventromedial prefrontal cortex (vmPFC) activity. Adequate integration of reinforcement history favors long-term advantageous decision-making (DM). Depressed patients might be influenced by immediate reinforcers, such as high rewards during a DM task including the IGT. After encountering a large reward a subsequent and unexpected penalty in addition to the absence of the presumably expected win might be associated with excessive habenula activation. Increased firing of the habenula results in low striatal dopamine and subsequently decreases vmPFC activation. This might then contribute to improving DM strategies and to a better outcome of the disorder. A specific subgroup of depressed patients with anhedonic symptoms might expect an inevitable punishment after obtaining a large reward during the DM task. Consequently the excessive firing of the habenula might not occur, leading to a relative increase in vmPFC activity. Dysfunction of the overactivated vmPFC affects DM strategies and is associated with “myopia” for the future as well as symptomatic persistence.

Historically, the IGT was a pioneering method in the examination of lesions of the vmPFC. Patients with bilateral damage to the vmPFC develop severe impairments in social and personal DM, otherwise having largely preserved intellectual abilities. These patients are characterized by “myopia” for the future, repeatedly engaging in decisions with long-term negative consequences in spite of previous experiences (Bechara et al., 1994). Structural and functional alterations of the vmPFC have long been implicated in the etiology of depression (Drevets et al., 2008). However, the exact role of the vmPFC and its interconnected subregions is not clearly understood (Myers-Schulz and Koenigs, 2012). Imaging studies report abnormally high levels of resting-state activity within the vmPFC in major depression (Greicius et al., 2007) potentially resulting in an altered DM process. An influential model of the role of vmPFC in affective disorders emphasizes the top-down inhibition of the amygdala and consequent control of the ventral tegmental area (VTA) dopaminergic neurons (Price and Drevets, 2010). When assessing the neuroanatomical correlates of DM and particularly reward processing, attention has more recently been directed to the habenular complex. Increased activity of the habenula has been implicated in the etiology of major depression (Shumake and Gonzalez-Lima, 2003). Functional hyperactivity of the habenula results in the suppression of dopamine cell activity in the VTA and subsequently, inhibition of the striatum, amygdala, nucleus accumbens, and prefrontal cortical areas, including the vmPFC (Hikosaka et al., 2008). The habenula plays a crucial role in behavioral responses to decisional consequences. In the absence of an expected reward increased activity of the habenula occurs (Hikosaka, 2010). After encountering a large reward during the IGT a subsequent and unexpected penalty in addition to the absence of the presumably expected win might be associated with habenula activation. An excessive firing of the habenula would result in low striatal dopamine and subsequently decrease vmPFC activation. However, we might speculate that some depressed patients rather tend to expect an inevitable punishment after obtaining a large reward during the IGT. Thus the excessive firing of the habenula might not occur. This in turn would lead to a relative increase in vmPFC activity in particular as a reaction to larger rewards (**Figure 1**). Strikingly, anhedonia has been associated with an excess of activity of the ventral region of the prefrontal cortex including the vmPFC, with a significant role of dopamine (Gorwood, 2008) Anhedonia in depression might be associated with a distinct pattern of regulation between interconnected neuroanatomical correlates. This is then manifested in a DM deficiency as measured by the IGT having a specific underlying mechanism.

### THE PREDICITING EFFECT OF A PERSISTENT DM DEFICIT ON DAILY LIFE AND SOCIAL INTERACTIONS

Depressed patients with anhedonic symptoms are characterized by reduced ability to modulate their DM strategies as a function of reward. They repeatedly opted for disadvantageous choices, disregarding long-term consequences. Furthermore, this DM tendency associated with anhedonia had a predictive value for symptom persistence and outcome in major depression (Vrieze et al., 2013). A neuroanatomical correlate of this deficiency is presumed to be the abnormally increased activity of the vmPFC resulting in functional alteration. Strikingly, depressed patients more responsive to antidepressive treatment exhibit a decrease in activation of vmPFC areas after medication is administered (Drevets et al., 2002). Similarly, depressed patients showing symptomatic remission to deep brain stimulation also exhibit a decrease in the activation of a vmPFC subregion after therapy (Mayberg et al., 2005). This suggests an association between the occurrence or reduction of clinical symptoms and the activity of specific brain areas. Furthermore, the regulatory processes between these interconnected brain structures might be reflected in DM strategies as measured by the IGT. Parallel to a decline in vmPFC activation patients tend to be influenced more by aversive stimuli (Koenigs and Grafman, 2009). An excess of vmPFC activity relates to a disturbance in reward learning manifested in the preference for immediate reward disregarding future consequences, i.e., “myopia” for the future. Thus, the opposite condition, i.e., a decrease in activation, might be the one beneficial on long-term leading to more advantageous choices. Of critical importance is the assumption that a more preserved DM strategy with intact reward learning is associated with correct value recognition in social life, supportive of remission (Zhang et al., 2012).

## CONCLUDING REMARKS

This targeted review aimed to direct special emphasis on the influence of anhedonic symptoms, the role of habenula and the vmPFC in dysfunctional DM and their combined effect on outcome prediction in depression. We propose that depressed patients with anhedonic symptoms tend to expect an inevitable punishment after obtaining a large reward during the IGT. Thus an excessive firing of the habenula typically detected in the absence of an expected reward does not occur. A consequential relative increase in vmPFC activity would then lead to dysfunctional DM strategies, disadvantageous choices, and a reduced ability to modulate behavior in the light of previous experiences. Disturbed reward responsiveness and reinforcement processing in association with anhedonic symptoms affect persistence of clinical symptoms and value recognition in everyday social life thus predicting outcome in depression (**Figure 1**).

The above concept integrating clinical markers, cognitive strategies and neuroanatomical correlates serving outcome prediction in major depression is targeted and by far not exclusionary. Another mechanism of significant importance involves the glutamatergic – GABAergic imbalance reflected by altered prefrontal levels of GABA and glutamate during value-guided choices reported in patients with major depressive disorder (Jocham et al., 2012). Future dedicated studies will not only favor clinical and neurocognitive research in depression but also assist clinical practice in treatment and outcome prediction.

## Conflict of Interest Statement

The authors declare that the research was conducted in the absence of any commercial or financial relationships that could be construed as a potential conflict of interest.
